# Supplementary material for: Drug repurposing for aging research using model organisms
Source: Aging Cell. 2017 Jun 16;16(5):1006–15. doi: 10.1111/acel.12626 (PMC5595691; doi:10.1111/acel.12626)
Supplement: Supplementary file 7 — Data S1 Zip‐Archive of all report cards. [file ACEL-16-1006-s007.zip › RC_SR7.pdf]

SR7

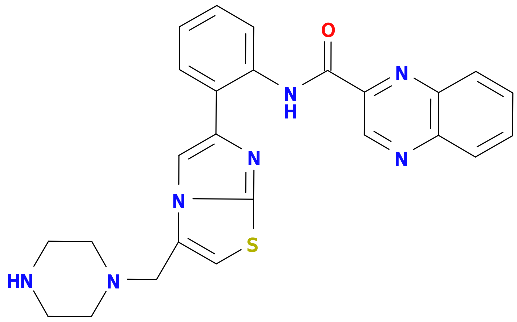

Database identifiers
ChEMBLCompound CHEMBL257991

Ranking

	Rank	Score
Drosophila	285/697	0.581
C. elegans	NA	NA

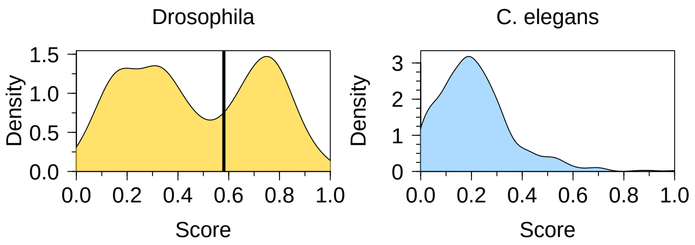

	Ageing implication	Domain conservation	Binding site conservation	Binding affinity	Bioavailability	Lipinski	Promiscuity	Purchasability	Drug approval	**Total**
Drosophila	1.0	0.74	0.95	0.919	(0.9)	0.0	-0.0	0.0	0.0	0.581
C. elegans	NA	NA	NA	NA	NA	NA	NA	NA	NA	NA

Names

No synonyms found

Roles

ChEBI entry None has no roles

Status

Approved drug (according to ChEMBL)	No
Number of Rule of 5 violations	0
Binding affinity to original target in log units (RF-Score prediction)	7.43
Burns *C. elegans* bioavailability prediction	9.23

Compound Target Characteristics

NAD-dependent protein deacetylase sirtuin-3, mitochondrial

Best gene implication in ageing for this target family came from gene Q9I7I7 annotated in UniProt release 2014_02. Annotation GO 8340 (determination of adult lifespan) was Inferred from Mutant Phenotype

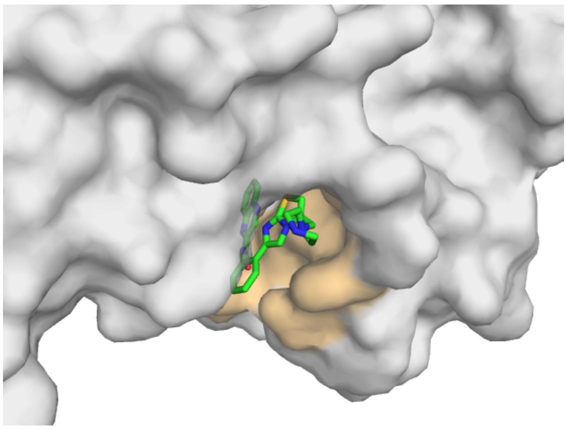

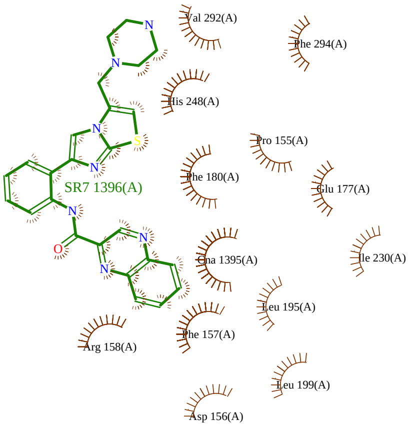

protein	amino acids contacts (binding site)											
PDB:4bn5:chainA:Q9NTG7	P	D	F	R	E	F	L	L	I	H	V	F
tr:E9PK80:E9PK80_HUMAN	P	D	F	R	E	F	L	L	I	H	V	F
sp:Q9NTG7:SIR3_HUMAN	P	D	F	R	E	F	L	L	I	H	V	F
tr:B2RZ31:B2RZ31_RAT	P	D	F	R	E	F	L	L	I	H	V	F
tr:C6ZII9:C6ZII9_RAT	P	D	F	R	E	F	L	L	I	H	V	F
tr:Q4FJK3:Q4FJK3_MOUSE	P	D	F	R	E	F	L	L	I	H	V	F
tr:D3YTK6:D3YTK6_MOUSE	P	D	F	R	E	F	L	L	I	H	V	F
sp:Q8R104:SIR3_MOUSE	P	D	F	R	E	F	L	L	I	H	V	F
sp:Q9I7I7:SIRT2_DROME	P	D	F	R	T	F	L	L	I	H	V	F
sp:P53686:HST2_YEAST	P	D	F	R	E	F	L	L	I	H	V	F

	whole protein		domain-based		contact-based	
protein	ident	simil	ident	simil	ident	simil
PDB:4bn5:chainA:Q9NTG7	1.0	1.0	1.0	1.0	1.0	1.0
tr:E9PK80:E9PK80_HUMAN	0.8	0.8	0.98	0.98	1.0	1.0
sp:Q9NTG7:SIR3_HUMAN	1.0	1.0	1.0	1.0	1.0	1.0
tr:B2RZ31:B2RZ31_RAT	0.55	0.62	0.79	0.89	1.0	1.0
tr:C6ZII9:C6ZII9_RAT	0.63	0.74	0.86	0.96	1.0	1.0
tr:Q4FJK3:Q4FJK3_MOUSE	0.55	0.62	0.79	0.89	1.0	1.0
tr:D3YTK6:D3YTK6_MOUSE	0.48	0.55	0.65	0.72	1.0	1.0
sp:Q8R104:SIR3_MOUSE	0.62	0.72	0.85	0.96	1.0	1.0
sp:Q9I7I7:SIRT2_DROME	0.34	0.55	0.46	0.7	0.92	0.95
sp:P53686:HST2_YEAST	0.26	0.49	0.35	0.64	1.0	1.0

Sirt2 (FBgn0038788) associated phenotypes

heat sensitive, lethal - all die before end of pupal stage, some die during pupal stage

(Information from FlyBase)

Sirt2 (UniProt:Q9I7I7) annotation

Function: NAD-dependent protein deacetylase (By similarity). May be involved in the regulation of life span. (, PubMed:17159295).
Cofactor: Zn(2+)Note=Binds 1 zinc ion per subunit. ;
Disruption phenotype: Causes lethality during development. Induced silencing shortens life span. (PubMed:17159295).

(Information from UniProt)
